# Spontaneous renal artery dissection presenting as an aortic dissection: a case report

**DOI:** 10.1186/s13256-016-1153-7

**Published:** 2016-12-20

**Authors:** Joshua Bucher, Ann-Jeanette Geib

**Affiliations:** Department of Emergency Medicine, Rutgers – RWJMS, 1 RWJ Plaza MEB 104, New Brunswick, NJ 08901 USA

**Keywords:** Renal artery dissection, Vascular dissection, Vascular surgery, Intensive care medicine, Aortic dissection, Case report

## Abstract

**Background:**

Renal artery dissection is a condition that has been associated with traumatic injuries and connective tissue disorders. It has been managed in the past by multiple methods because there is no standard treatment, including vascular intervention with angioplasty and stenting, anticoagulation/antiplatelet therapy, and hypertension management.

**Case presentation:**

We present a case of a spontaneous renal artery dissection in a 55-year-old white man with no traditional risk factors who presented twice to our emergency department in a 2-day period with different symptoms; on his first presentation he presented with symptoms consistent with renal colic and on the second visit he presented with symptoms consistent with aortic dissection.

**Conclusions:**

Our patient was treated with endovascular stent placement by interventional radiology, heparin infusion, and admission to our medical intensive care unit. Our review here highlights the varied management of this diagnosis for which there is no standard treatment and decisions are made in conjunction with consultants.

## Background

Renal artery dissection has been infrequently reported in the literature. It has been associated with blunt traumatic injury and connective tissue disorders [1]. There is no standard treatment for this condition as the literature reports multiple different treatment methods with varying outcomes. We present a case of a patient with a spontaneous renal artery dissection in the absence of any traditional risk factors.

## Case presentation

Our patient was a 55-year-old white man who presented to our emergency department (ED) with a 2-day history of severe, dull, constant left flank pain with radiation to his groin, and nausea with no vomiting. He denied hematuria, fever, chills, dysuria, or discharge from his penis. He denied any history of renal colic, chest pain, shortness of breath, or any recent traumatic injury.

His past medical history was pertinent for psoriasis, tympanostomy tubes during childhood, cholecystectomy, and temporomandibular joint (TMJ) pain. He took adalimumab (Humira™) for psoriasis and was recently prescribed prednisone for his TMJ pain. His only allergy was to penicillin. He specifically denied any history of renal disease.

His initial vital signs were 36.2 °C (97.2 °F), 64 beats per minute (bpm), 18 respirations per minute (rpm), blood pressure (BP) 150/89, and blood oxygen saturation (spO_2_) 99%. On examination, he was in moderate painful distress. His skin was warm and dry and he did not have any diaphoresis. The only positive findings on his abdominal/back examination was left upper quadrant tenderness and left flank/costovertebral angle (CVA) tenderness.

His white blood cell (WBC) count was 15.6 with 85% neutrophils and no band neutrophils. His electrolytes were unremarkable, but he had blood urea nitrogen (BUN) of 21 and creatinine (Cr) of 1.6 with no history of renal disease. (See Table 1 for pertinent laboratory values.) A urine analysis was normal. A computed tomography (CT) scan without contrast was done to assess for renal stones; it was interpreted as normal per radiology.

**Table 1:** Laboratory values on day prior to diagnosis and day of diagnosis

Day prior to diagnosis		Day of diagnosis	
White blood cell	15.6 × 10^9^/L	White blood cell	17.7 × 10^9^/L
Hemoglobin	15.6 g/dL	Hemoglobin	15.4 g/dL
Blood urea nitrogen	21 mg/dL	Blood urea nitrogen	26 mg/dL
Creatinine	1.6 mg/dL	Creatinine	1.7 mg/dL
Potassium	4.6 mEq/L	Potassium	5.0 mEq/L
Bicarbonate	21.7 mEq/L	Bicarbonate	21.1 mEq/L

He received fluids administered intravenously, hydromorphone, ketorolac, and ondansetron and felt significantly improved. He was discharged home with prescriptions and follow-up instructions for abdominal pain and instructed to return if his condition worsened. He returned to our ED the following day. On arrival, he complained of 2 hours of “crushing” midsternal chest pain described as an “elephant sitting on my chest” with “ripping” radiation from his front to this back associated with diaphoresis. He was uncomfortable and in severe painful distress and could not cooperate with the medical examination or with obtaining an electrocardiogram (ECG).

He initially received 3 mg of IV hydromorphone administered intravenously in order to evaluate him and obtain an ECG, which was normal. His vital signs were 35.9 °C (96.7 °F), 83 bpm, 16 rpm, BP 156/95 in both arms, and spO_2_ 100% on room air (RA). He was profusely diaphoretic but his examination was otherwise unremarkable. Of note, he no longer had flank or abdominal tenderness.

He was taken immediately for a CT angiogram (CTA) of his chest/abdomen/pelvis to evaluate for aortic dissection. On laboratory diagnostics, he had a WBC count of 17.7 with 83% neutrophils, a normal troponin level, BUN and Cr of 26 mg/dL and 1.7 mg/dL, and a potassium level of 5.0 mEq/L (see Table 1 for additional diagnostics). His CTA was interpreted by a radiologist as: “a segment of proximal left renal artery demonstrating significant focal stenosis/near occlusion, with severely delayed left nephrogram and only minimal left renal parenchymal enhancement…(the) stenosis appears to represent asymmetrical mural thickening and could represent an eccentric thrombus or focal thrombosed dissection”.

On review of the CTA results with the attending radiologist, the departments of interventional radiology (IR) and vascular surgery were both immediately consulted. Our patient was taken to the IR suite for acute intervention. Under fluoroscopic guidance, it was discovered that his left renal artery had dissected, and a stent was placed. A heparin infusion was initiated and he was admitted to our medical intensive care unit (MICU) for further monitoring. The rest of his hospital course was unremarkable with some improvement in his renal dysfunction. A transesophageal echocardiogram was negative for aortic dissection. He was discharged home after 4 days.

## Discussion

Spontaneous, isolated renal artery dissection is a condition that is infrequently reported in the literature. It is thought to be associated with connective tissue disorders, such as Ehlers–Danlos syndrome, hypertension, and aortic dissection with secondary dissection into the renal arteries [1]. One study performed at the University of Michigan compared characteristics of 17 patients who were diagnosed as having a spontaneous renal artery dissection [1]. The most common complaint was sudden onset excruciating flank pain ipsilateral to the site of the dissection, similar to our patient’s initial presentation. Nine of the 17 patients had a connective tissue disorder or autoimmune connective tissue disorder. The patients were more likely to have hypertension, cancer, and connective tissue disorders, and less likely to be obese than the general population [1].

A literature search was unable to find any relationship or case reports of patients taking adalimumab and an arterial phenomenon such as dissection or thrombosis. However, the prescribing drug information did note that there was one case of “thrombosis leg,” although it did not provide details of the patient’s reaction [2]. There have been reports of patients with arterial phenomena such as a dissection associated with steroid use. There is a case report of a patient on long-term prednisone with catheter-induced dissection of a coronary artery [3]. Another case of a spontaneous rupture of a pulmonary artery was also seen in a patient with chronic prednisone use [4]. Our patient was taking stress-dose steroids for a short period of time. There may be a temporal relationship with the steroids.

Due to the infrequent presentation of spontaneous renal artery dissection, a consensus for treatment has not been reached. Several cases had endovascular treatment with balloon angioplasty, stenting, or both [5–7]. Other cases involved medical treatment such as antihypertensive medications, pain control, and anticoagulation or antiplatelet agents. One case series of four patients, three of whom had dissections treated with conservative medical management, found that at 1-month follow-up after treatment with enoxaparin and aspirin, all three patients treated with medical management had normal kidney function, normal blood pressure, and had revascularization of the majority of their kidneys [8]. Another two cases of spontaneous renal dissection, which had only caused ischemia to a fraction of the kidney or were not associated with ischemia, were both successfully treated with conservative medical management involving anticoagulation and antiplatelet agents with a good outcome [9, 10].

This case has several important considerations for emergency physicians. Since there is no standard care for this condition, consultation with the appropriate specialist should be obtained to discuss the optimal treatment. In the ED, the patient should be prepared for surgery or any interventional procedure, with a type and screen and coagulation factors ordered. The patient should be kept without oral intake prior to any procedure.

Consultation with a vascular surgeon, interventional radiologist, or nephrologist is recommended or a transfer to a facility with an available specialist for evaluation if none are available at your facility is indicated. The literature, as noted above, described multiple different treatment methods, including anticoagulation, antiplatelet agents, stenting, and aggressive blood pressure control without other intervention. Due to the multiple different treatments described, the optimal treatment is unknown.

The patient will probably require admission to a monitored environment for frequent reevaluation for worsening symptoms or progression of the dissection.

## Conclusions

At our facility, IR ultimately placed a stent into our patient’s renal artery and infused intra-arterial tissue plasminogen activator (tPA). He was also started on anticoagulation treatment that was continued after discharge from our facility. He ultimately recovered from his renal dysfunction and on renal ultrasound 2 weeks later he had improved flow to his affected kidney.

## References

[1] Afshinnia F, Sundaram B, Rao P, *et al*. Evaluation of characteristics, associations and clinical course of isolated spontaneous renal artery dissection. Nephrol Dial Transplant. 2013;28(8):2089–98. 10.1093/ndt/gft073 PMC3888135 23563282

[2] Highlights of Prescribing Information. Humira. http://www.rxabbvie.com/pdf/humira.pdf. Published 2002. Updated 9/2013. Accessed 18 Apr 2014.

[3] Miyachi H, Tanaka K, Mizuno K. Catheter-induced bilateral coronary ostium dissection in a patient with long-term steroid therapy. J Invasive Cardiol. 2012;24(11):E305–7. 23117328

[4] Zenker D, Fuzesi L, Aleksic I. Spontaneous rupture of the left pulmonary artery – caused by long-term steroid use? Eur J Cardiothorac Surg. 2003;24(6):1037–9. 10.1016/s1010-7940(03)00576-1 14643832

[5] Tandon G, Sukhija R. Isolated spontaneous renal artery dissection: a case report and review. Int J Angiol. 2012;21(2):99–102. 10.1055/s-0032-1315623 PMC3444006 23730138

[6] Casciani E, Polettini E, Masselli G, Stirati G, Gualdi G. Spontaneous renal artery dissection diagnosed by unenhanced magnetic resonance angiography: case report. Urol Int. 2012;89(4):486–8. 10.1159/000339751 22777312

[7] Shmorgun D, Claman P, McGregor P, *et al*. Renal artery dissection during an *in vitro* fertilization/intracytoplasmic sperm injection cycle. Fertil Steril. 2009;92(4):1498.e1–3. 10.1016/j.fertnstert.2009.06.045 19709654

[8] Abdel-Kerim A, Cassagnes L, Alfidja A, *et al*. Management of isolated non-traumatic renal artery dissection: report of four cases. Acta Radiol. 2012;53(4):401–5. 10.1258/ar.2012.110303 22517982

[9] Hsieh MJ, Lin YH, Liu KL. Spontaneous renal artery dissection. Emerg Med J. 2012;29(12):1006. 10.1136/emj.2009.074732 21068175

[10] Misraj V, Peyromaure M, Poiree S. Spontaneous dissection of branch renal artery—is conservative management safe and effective? J Urol. 2006;176(5):2125–9. 10.1016/j.juro.2006.07.017 17070275

